# Risk Factors for Concussion in Under 18, Under 22 and Professional Men's Rugby Union: A Video Analysis of 14,809 Tackles

**DOI:** 10.1186/s40798-023-00642-z

**Published:** 2023-10-14

**Authors:** Takayuki Kawasaki, Yuta Kawakami, Shuko Nojiri, Yoshinori Hasegawa, Manabu Kuroki, Shogo Sobue, Kenta Shibuya, Yuji Takazawa, Muneaki Ishijima

**Affiliations:** 1 Department of Orthopaedics Surgery, Faculty of Medicine, Juntendo University, 2-1-1, Bunkyo, Tokyo, 113-8421 Japan; 2 Department of Mathematics, Physics, Electrical Engineering and Computer Science, Graduate School of Engineering Science, Yokohama National University, Kanagawa, Japan; 3 Department of Medical Technology Innovation Center, Juntendo University, Tokyo, Japan; 4 Division of Intelligent Systems Engineering, Faculty of Engineering, Yokohama National University, Kanagawa, Japan; 5 Faculty of Health and Sports Science, Juntendo University, Chiba, Japan

**Keywords:** Rugby, Concussion, Tackle, Video analysis

## Abstract

**Objective:**

This study aimed to identify the risk factors for tackle-related concussion observed in matches involving under (U) 18, U 22 and professional men’s Rugby Union players through video analysis.

**Study Design:**

Descriptive epidemiology study.

**Methods:**

Twenty Rugby Union matches each for high school (U18), university/college (U22) and professional (Elite) were randomly selected from 202 matches in the 2018/2019 season. Both one-on-one and tackles involving multiple tacklers were analyzed for the 60 matches. The 28 categorical and continuous variables (e.g., tackle characteristics and duration before the tackle) were applied as risk factors to a least absolute shrinkage and selection operator (Lasso) regression analysis. To identify high-risk situations, a simulation model with coefficients obtained from the Lasso regression was used. Statistical analysis was conducted according to tackle direction.

**Results:**

A total of 14,809 tackles and 41 concussions involving 1800 players were included in the analyses. The incidence rate of concussions (injuries/1000 tackles) was greater in Elite players (4.0) compared with U18 (1.9) and U22 (2.4) players. The factors most highly associated with concussions were head-in-front tackles (where the tackler’s head is placed forward, impeding a ball carrier’s forward movements, 11.26/1000 tackles), and were more often observed among U18 players. A simulation model predicted that the highest risk tackle situation in Elite players was a head-in-front, side-on tackle below the hip of the ball carrier (predicted incidence rate 18.07/1000 tackles).

**Conclusion:**

The risk factors associated with concussion need to be assessed cautiously. Avoiding head-in-front, side-on tackles to the lower extremities of a ball carrier should be considered to reduce injury risks.

**Supplementary Information:**

The online version contains supplementary material available at 10.1186/s40798-023-00642-z.

## Key Points

• This video analysis study assessed 14,809 tackles to identify risk factors for concussions.

• Although the incidence rate of concussion was higher in professional rugby players, high school players tended to employ more head-in-front tackles that were highly associated with the risk of concussion.

• A high-risk tackle situation was predicted when a professional player employed a head-in-front tackle to the lower extremities of the ball carrier in side-on tackle situations, suggesting that lowering tackle height does not necessarily reduce injury risk.

**Supplementary Information:**

The online version contains supplementary material available at 10.1186/s40798-023-00642-z.

## Introduction

Rugby Union requires fast movements that often coincide with collisions, frequently exposing players to traumatic injuries, including concussion [1]. Long-term outcomes after concussion in collision athletes have raised safety concerns [2]. In the UK, it has been proposed that the tackle should be removed from Rugby Union in schools as a means of reducing the risk of injuries. However, it remains unclear whether repeated head impacts (e.g., concussions) experienced at a young age may affect players later in life. Moreover, such a proposal would inevitably change the nature of Rugby Union and may increase the risk of later injury [3].

Recent studies reported that a tackle event is the most common cause of concussion, and 70% of tackle-related concussions occur in the tacklers [1, 4, 5]. Detailed analysis of tackles using video footage has become a standard method for investigating the cause of the injuries [4–9], and analyzing the body position and height of the tackle may be of value for reducing tackle-related concussion [1, 4, 5, 10–17].

High tackles, which are defined as a hit above the line of the nipple of the ball carrier, can be a risk factor for concussion [1, 4, 5] and thus were banned in 2018. On the other hand, a video analysis study in New Zealand reported that players making low tackles have a higher risk of head injury [10]. The association between the height of tackles and the risk of head injuries is complex and remain unclear [15–17]. Previously, our group reported that a tackle where the tackler’s head is placed forward, impeding a ball carrier’s advance movements (*head-in-front tackle*), resulted in 30 times higher risk of head, neck, and shoulder injuries than those with a correct head position [13]. However, this study was limited to two university teams and the analysis was conducted with the head positions of the tacklers.

Tackle-related injuries appear to be affected by poor tackling technique, not by intention  [11]. Youth and non-professional players may have poorer tackle techniques compared with the professional players. Hence, it is reasonable to investigate detailed tackling characteristics stratified by age and professional levels (i.e., U18, U22, and Elite). Evaluating tackle height and head position in these subgroups may indicate the risk factors for tackle-related injuries, particularly concussions and may be ﻿useful in strategies to prevent injuries.

This study aimed to identify the risk factors for concussion associated with the types and characteristics of tackles as well as playing situations, e.g., head position, tackle height, tackle direction, player’s position, area, and period of play. Using video clips from professional, university/college (U22) and high school (U18) men’s Rugby Union matches, we analyzed the tackles related to concussions.

## Methods

### Participants

This study was conducted using video clips from 202 matches in men’s Rugby Union competitions over the 2018/2019 season (50 matches in the National High School Rugby competition [U18], 72 matches in the National University/College competition [U22], and 80 matches in the Japan Rugby Top League competition [the present League One]). The study protocol was approved by the local ethics committee (approved number: 2019069), and permission was obtained by Japan Rugby Football Union for use of video clips. This study was performed in accordance with the standards of ethics outlined in the Declaration of Helsinki.

### Procedures

Of 202 Rugby Union matches in high school (U18), university/college (U22) and professional (Elite) rugby players, 20 matches each were randomly selected by using a random number generator. Both one-on-one tackles and those involving multiple tacklers (player or players making the tackle) ﻿and a ball carrier (player or players carrying the ball) ﻿were analyzed in the total of 60 matches. When a tackle occurred between a ball carrier and more than one player, the tackler who made first contact with the ball carrier was included in the analysis. A tackle was defined as “any event where one or more tacklers attempted to stop or impede the ball carrier, whether or not the ball carrier was brought to the ground” [18]. World Rugby defined tackle type as follows: shoulder tackle front-on; shoulder tackle side-on; smother tackle, tap tackle; tackles involving more than one tackler; tackle from behind; tackling in a dangerous manner (penalty). These describe the appearance of the tackle, but do not mention the height or head position of the tackler. However, the latter are reported as risk factors for concussion [13, 17]. In this study, we assessed the tackles in line with the previous studies [6, 11, 14, 16, 19, 20] and categorized them by the head position of a tackler toward a ball carrier (correct or head-in-front position); direction of tackle (front-on, side-on, behind); height (chest, torso, below the hip). Twenty-three categorical and five continuous variables were applied to identify intrinsic and extrinsic risk factors for concussions (Additional file 1: Table S1).

Illegal tackles including high tackles were excluded from the analysis due to high possibility of head injuries, leading results to be biased. Continuous variables were applied to assess whether the playing situation (being chaotic and complex) and player emotion (due to winning or losing situations) affect the risk of concussion. The score difference and final score difference between teams when a tackle occurred were assessed using the following equation:

$${\text{Score}}\;{\text{difference}} = \left[ {{\text{Score}}\;{\text{of}}\;{\text{the}}\;{\text{tackler}}^{{\prime }} {\text{s}}\;{\text{team}}} \right]{-}[{\text{Score}}\;{\text{of}}\;{\text{ball}}\;{\text{carrier}}^{{\prime }} {\text{s}}\;{\text{team}}]$$

Thus, a positive score indicated the tackler in the winning situation and a negative score indicated the tackler in the losing situation. Duration 1 was defined as the duration from a starting phase (scrum, lineout, maul, ruck, etc.) until the next tackle, and Duration 2 was defined as the duration between the moment the player last obtained the ball and the time of being tackled. Descriptions of tackle types and characteristics and other factors analyzed in this study are available in Additional file 1: Table S1. Tackles were evaluated by three professional analysts (out of the authors) using the Rugby Analyzer (DataStadium Inc., Tokyo, Japan), where each tackle was numbered, and the examinee rated the characteristics of a tackle under conditions in which rewind and slow playback were freely permitted. For consistency and accuracy of analysis, three of 60 game video clips (5%) were randomly selected and preliminarily analyzed by three professional analysts. Inter-rater reliability, which was assessed with percentage agreement (kappa coefficient) of the head position, direction, and tackle type, were 96.3% (0.85), 90.7% (0.84) and 90.2% (0.85), respectively. Subsequently, further analysis of each of the video clips from the 20 matches was undertaken by these three analysts to analyze all of the 60 matches. When concussion was observed in the video, this was confirmed using the medical records of each match that were recorded by the team doctor and the match day doctors.﻿﻿

### Statistical Analysis

Poisson distribution was applied as injury events per 1000 tackles, which is commonly used to describe injury incidence rate for a rare event. Least absolute shrinkage and selection operator (Lasso) for Poisson regression analysis was used. The continuous variables were used as unconverted, and the categorical variables were converted to dummy-variables (0, 1) for analyses.

All variables were included as covariates to evaluate the effect of the risk factors for concussion, and all variables other than tackle characteristics were included to evaluate the risk ratio of a head-in-front tackle. The Lasso regression analysis was conducted for front-on tackles, side-on tackles and all directions of tackles, and behind tackles were excluded as there was only one injury event observed for behind tackles. The regularization parameter (ℷ) was chosen to minimize the Poisson deviance by a tenfold cross-validation method using R packages (glmnet). The incidence rate ratio (IRR) was evaluated using the exponent of the slope obtained. After obtaining all the coefficients from the Lasso analysis, we predicted the concussion incidence rate by assigning specific values (i.e., mean, dummy value) into variables of the Lasso regression model for each tackle situation. The categorical variables considered in this simulation model were: the level of the players, head position of the tackle and tackle height. Statistical analysis was performed using the free software R (The R Foundation for Statistical Computing, Vienna, Austria).

## Results

### Population Analysis

A total of 1800 players were involved in this study, with participant demographics in Additional file 1: Table S2. The mean (standard deviation, SD) ages of U18, U22 and Elite players were 17.4 (0.7), 20.8 (1.1) and 27.8 (3.5), respectively. Of a total of 14,853 tackles analyzed, 44 illegal tackles that resulted in no injury were excluded from the analysis. In particular, 34 (75%) of the tackles excluded were high tackles, while the remainder were considered to be ‘dangerous tackles’. Therefore, a total of 14,809 tackles were included in the analysis. Of these, 4718, 5345 and 4746 tackles and 9, 13 and 19 injuries were observed in the U18, U22 and Elite players, respectively (Table 1). The injury incidence rate was numerically greater in Elite players (0.4%) compared with U18 (0.19%) and U22 (0.24%) players. However, the highest percentage of head-in-front tackles occurred in the U18 players. A higher number of smother tackles were observed in the Elite players (29.7%) compared with U18 players (21.1%).

**Table 1:** Number of tackles associated with injuries and tackle characteristics by player levels

	U18	U22	Elite	Total
Number of tackles	4718 (100%)	5345 (100%)	4746 (100%)	14,809 (100%)
*Concussion*	9	13	19	41
*Incidence, injury events/1000 player hours (95% CI)*	15.0 (5.6–24.4)	16.3 (5.3–27.2)	23.8 (8.3–39.2)	18.3 (11.6–25.0)
*Incidence, injury events/1000 tackles (95% CI)*	1.91 (0.66–3.15)	2.43 (1.11–3.75)	4.00 (2.21–5.80)	2.77 (1.92–3.62)
Tackle characteristics				
*Head placement on the side of the ball carrier*				
Correct head position	3968 (84.10%)	4613 (86.30%)	4264 (89.84%)	12,845 (86.73%)
Head-in-front position	750 (15.90%)	732 (13.70%)	482 (10.16%)	1964 (13.26%)
*Direction*				
Front-on	2709 (57.42%)	3002 (56.16%)	2630 (55.42%)	8341 (56.32%)
Side-on	1676 (35.52%)	1967 (36.80%)	1665 (35.08%)	5308 (35.84%)
Behind	333 (7.06%)	376 (7.03%)	451 (9.50%)	1160 (7.83%)
*Tackle height*				
Chest	2034 (43.11%)	2361 (44.17%)	2596 (54.70%)	6991 (47.21%)
Torso	2153 (45.63%)	2454 (45.91%)	1745 (36.77%)	6352 (42.89%)
Below the hip	531 (11.25%)	530 (9.92%)	405 (8.53%)	1466 (9.90%)
*Tackle type*				
Shoulder	2660 (56.38%)	2926 (54.74%)	2205 (46.46%)	7791 (52.61%)
Arm	1064 (22.55%)	1229 (22.99%)	1130 (23.81%)	3423 (23.11%)
Smother	994 (21.07%)	1190 (22.26%)	1411 (29.73%)	3595 (24.28%)

Percentage is shown as a fraction of total number of tackles in each group as 100%.

### Factors Associated with Concussion

The number of concussions associated with tackle characteristics and other factors, and the injury incidence rate (injuries/1000 tackles) in front-on, side-on, and behind tackle situations are summarized in Table 2. Concussions were observed more frequently in the Elite group compared with the U18 and U22 groups, and the injury incidence rate was much higher in head-in-front tackles than correct head tackles, irrespective of the direction of the tackle.

**Table 2:**
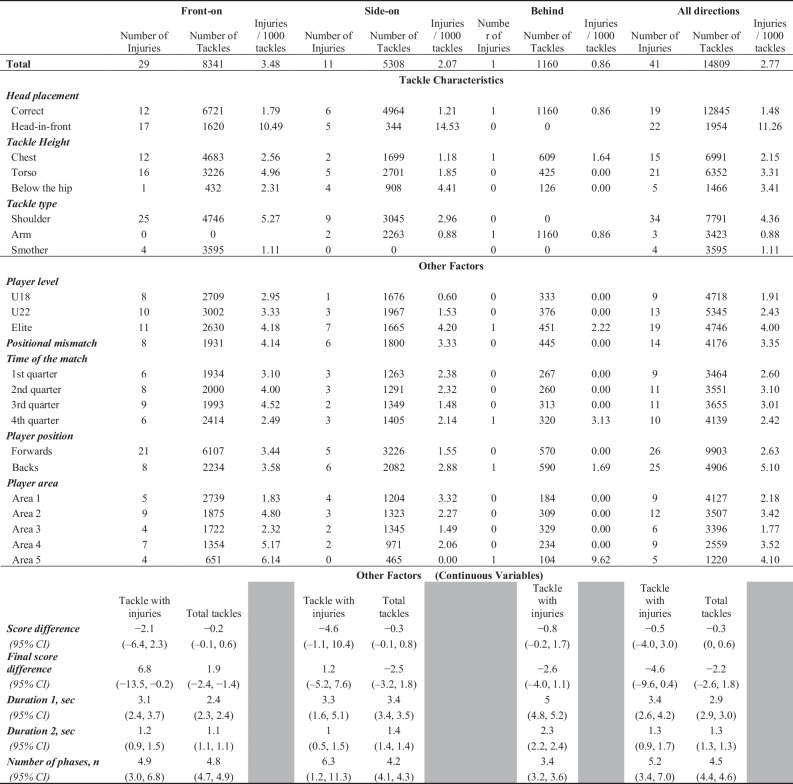
Injury incidence rate of the tackle and other factors analyzed in this study

Mean and 95% confidence interval (CI) are shown for the continuous variables.

Due to the many possible predictors as well as multicollinearity, we used the Lasso regression model that shrinks the less important variables to close to zero. Important factors evaluated to be related to the concussions are shown in Table 3. The IRR indicated the risk ratio in the factorial situation when compared with the non-factorial situation. For instance, head-in-front tackles were evaluated to have a 3.93-, 7.81- and 5.77-times higher risk for concussions than correct head tackles in the front-on, side-on, and all directions of tackles, respectively. Tackling below the hip of the ball carrier in side-on tackles was evaluated to have a 1.13-times higher risk for concussions when compared with chest or torso tackles. Elite players were evaluated to have a 1.78- and 1.36-times higher risk for concussions in side-on and all directions of tackles when compared with U18 or U22 players. However, the risk in front-on tackles was not significantly different between groups. Lasso analysis also showed that the risk of concussion would increase by 1.04- and 1.01-times when Duration 1 increased by one second in side-on and all directions of tackles. Similarly, it was evaluated that the risk of concussion would increase by 1.02-times for each additional phase of play in side-on tackles. (Table 3).

**Table 3:** Factors related to concussion using the Lasso regression analysis

Incidence rate ratio	Front-on tackle	Side-on tackle	All directions
Head-in-front	3.93	7.81	5.77
Below the hip		1.13	
Elite		1.78	1.36
Area 1	0.82		
Duration 1	1.04		1.01
Number of phases		1.02	

### High-Risk Tackle Situations Predicted by the Simulation Model

The injury incidence rate was calculated using the Lasso regression model to predict high-risk situations, and 36 distinctive combinations of factors were assessed. A head-in-front tackle in front-on tackle situations had a 3.93-times higher risk for concussions than correct head tackles, regardless of player levels and tackle height. However, head-in-front tackles that occurred below the hip of the ball carrier in side-on tackles were predicted to have a higher risk when compared with chest or torso tackles. The predicted injury incidence rate for a head-in-front tackle in side-on situations was 7.81 times higher than a correct head tackle. The highest injury incidence rate (18.07 injuries/1000 tackles) was predicted when Elite players employed a head-in-front tackle below the hip in side-on tackle situations (Table 4).

**Table 4:** Injury incidence rate predicted using the simulation model

Tackle direction	Level	Head position	Tackle height	Predicted incidence rate (injuries/1000 tackles)
Front-on tackle	U18/U22/Elite	Head-in-front	Chest/torso/below the hip	9.31
	U18/U22/Elite	Correct	Chest/torso/below the hip	2.37
Side-on tackle	U18/U22	Head-in-front	Chest/torso	8.97
	U18/U22	Head-in-front	Below the hip	10.14
	Elite	Head-in-front	Chest/torso	15.97
	Elite	Head-in-front	Below the hip	18.07
	U18/U22	Correct	Chest/torso	1.15
	U18/U22	Correct	Below the hip	1.30
	Elite	Correct	Chest/torso	2.05
	Elite	Correct	Below the hip	2.31

### Factors Related to Risk Ratio of Head-in-Front Tackles

Using Lasso regression analysis, the players’ risk ratio to employing head-in-front tackles was assessed with many factors being presumed to be related to a head-in-front tackle. Similar to the results shown in Table 1, the risk ratio of head-in-front tackle was higher in U18 players than in Elite players in all directions of tackle analyzed (Table 5). The increased score difference and the final score difference incrementally affected the risk ratio of head-in-front tackles, although the effect was minimal (approximately 1.002 times more by increasing one score). The risk ratio of head-in-front tackles declined when Duration 2 (the duration between the moment the player last obtained the ball and the time of being tackled) increased. For the remaining factors, the risk ratio of head-in-front tackles differed between front-on and side-on tackle situations. In particular, forward players were less likely to use head-in-front tackles than backs (IRR, 0.56).

**Table 5:** The factors related to the risk ratio of head-in-front tackles

Incidence rate ratio	Front-on tackle	Side-on tackle	All directions
U18	1.13	1.11	1.17
Elite	0.75	0.98	0.77
Positional mismatch		1.15	0.95
1st quarter	1.05	0.99	1.00
2nd quarter		1.20	1.00
3rd quarter		0.93	0.95
Player position			
Forwards	0.96	0.56	0.92
Backs		1.00	1.00
Area 1	1.26		1.26
Area 2	1.02		
Area 3		1.10	
Area 4		0.76	0.95
Area 5	0.98		0.97
Score difference	1.00		1.00
Final score difference		1.00	1.00
Duration 1	0.98	1.05	0.95
Duration 2	0.95	0.96	0.88
Number of phases	1.00	0.99	1.00

## Discussion

### Main Findings

In this study, 14,809 tackles undertaken by 1800 players in U18, U22 and Elite teams were analyzed to identify the risk factors for concussion associated with the types and characteristics of tackles as well as playing situations. There were more concussions in Elite players, and head-in-front tackles may increase the risk of concussion, although the risk ratio of head-in-front tackles was greater in U18 players. The risk of concussion was different between front-on and side-on tackle situations, and the highest risk was predicted when Elite players employed head-in-front tackles below the hip of the ball carrier in side-on tackle situations.

The incidence rate of concussion was greater in Elite players than U18 or U22 players, which was consistent with the findings in other studies [7–9, 21]. It is likely due to the greater momentum gained by heavier, taller players with high speed. Increased weight and height in professional players compared with U18 and U22 players were confirmed using participant demographics (Additional file 1: Table S2).

This study illustrated the increased risk for concussion with head-in-front tackles compared with correct head tackles. This finding is consistent with a previous study [13]; however, the IRR reported in this study was lower (3.93–7.81), compared with the risk ratio of 30 reported in the previous study [13]. Two main reasons could explain this discrepancy. First, one-third of subjects in the present study were U18 players who often employed head-in-front tackles but had low incidence of concussions, whereas in the previous study, all players were U22 players. Second, we used the Lasso regression method in this study that shrinks the coefficients that were less related. It should be noted that the relative risk ratio based on the injury incidence rate of each factor would be different from the results of Lasso regression.

The Lasso regression analysis indicated that side-on tackles that occurred below the hip of the ball carrier had a higher risk for concussion compared with chest or torso tackles. However, the height of the tackle did not affect the risk of concussion in front-on tackle situations. Whether tackle height influences the risk of concussion is still controversial. Current regulations prohibit tackles above the shoulder of the ball carrier [18] and recent studies have suggested that tackles should be made below the chest to reduce the risk of concussions [12, 15, 16]. Tierney and Simms [16] also suggested that tackling on the upper trunk of the ball carrier and avoiding tackling to the upper legs would reduce the risk for concussions. The results of our study indicated that tackling below the hip of the ball carrier had a higher risk for concussions of the tackler, which is almost consistent with the findings of Tierney’s study. However, due to the different statistical analysis used, we demonstrated a higher risk of concussions with tackles that occurred below the hip in side-on tackles only, not in front-on tackles. This result was confirmed by the simulation model fitted with the data of this study.

### Interpretation of the Analysis and Implications

Although Elite players were at higher risk of concussion compared to U18 and U22 players, the one factor that had the greatest IRR was a head-in-front tackle, which suggests that head-in-front tackles could be the most critical risk factor for concussions. Our results demonstrated that head-in-front tackles were used more by the U18 players compared with Elite players. In addition, smother tackles were less prevalent in U18 players compared with Elite players. Considering that most (83%) of the concussions were observed in shoulder tackles (Table 2), we presume that smother, as well as arm tackles, could reduce the risk of concussion. However, coefficients of tackle type were greatly shrunk in the Lasso analysis as tackle type was highly correlated to head-in-front tackles and some injury event was zero leading the IRR unavailable. Increasing the risk ratio for U18 players to use smother tackles may play an important role in reducing the risk of concussion.

Many factors were related to head-in-front tackles (Table 5), and this is likely one of the main reasons why limited coefficients of factors for injury risk were identified in the Lasso analysis (Table 3). Fewer head-in-front tackles were employed by forwards compared with backs in side-on tackles. However, the IRR of head-in-front tackles for concussion in side-on tackles was greater when compared with correct head tackles, which is likely due to the high injury incidence rate in players in backs positions. It should be highlighted that U18 players had a higher risk ratio of head-in-front tackles, and this risk would be higher when players had a shorter time before the tackle (Duration 2) and when the score difference was greater. This study encouraged the necessity of training to reduce the risk for concussions, namely avoiding head-in-front tackles and using the smother tackle, particularly for U18 players, and avoiding head-in-front tackles below the hip of the ball carrier in side-on tackle situations. Previously, we reported that most of the head-in-front tackles occurred unintentionally [13]. Tierney et al. also demonstrated that Foot planting might compromise the tackler’s technique and timing, which may prevent the tackler from placing their head to the side of the ball carrier during the tackle [22].

Regarding the tackle height, in 2008, Quarrie et al. demonstrated that tacklers making low tackles resulted in a higher rate of injuries (not restricted to head injuries) per 1000 tackles (incidence rate [95% confidence interval]) 2.2 [1.5–3.3]) than those making high (1.1 [0.9–1.4]) or middle (1.9 [1.5–2.3]) tackles [10]. Moreover, in 2018, Tierney et al. demonstrated that intended primary contact at the upper trunk of the ball carrier led to more frequent Head Injury Assessments for the tackler [16]. They also mentioned that lowering the maximum legal tackle height to below the upper trunk of the ball carrier could reduce the risk of concussion. However, it also increased the needs for Head Injury Assessment when the tackle contacted the upper leg of the ball carrier. In conjunction with our results, lowering tackles would not necessarily reduce the risk of concussions. Further studies should be needed to explore these issues.

## Limitations

There are several limitations in the present study. Due to the nature of video analysis, severity of the concussion is unknown. We did not assess intra-rater reliability, the kinematics of the tackles or measure the velocity and force of the tackles as well as the characteristics of the ball carrier at the tackle event. In the current study, 44 illegal tackles, including high tackles, were excluded from the analysis. None of these 44 tackles resulted in any injury to the tackler. Consequently, we consider that the exclusion of these tackles would not substantially affect the results. However, the results might have been slightly different if they had been included. Although our results were shown to be in line with other studies, the rationale for the higher injury incidence rate in Elite players remains unclear.

## Conclusions

This large-scale video analysis study suggested that head-in-front tackles can be highly associated with concussions and that head-in-front tackles below the hip of the ball carrier in side-on tackles could also account for concussions. The results of our study support the evidence that lowering tackles would not necessarily reduce the risk of concussions and head-in-front tackles to the lower extremities of the ball carrier in side-on tackle situations should be avoided.

### Supplementary Information

**Additional file 1: Table S1**. Description of tackle characteristic and factors analysed in this study.** Table S2.** Participant demographics.

## Abbreviations

IRR: Incidence rate ratio
Lasso: Least absolute shrinkage and selection operator

## Declarations

### Data Sharing

De-identified individual participant data are available upon reasonable request from the corresponding author (TK).

## References

[1] Tucker R, Raftery M, Kemp S, Brown J, Fuller G, Hester B (2017). Risk factors for head injury events in professional rugby union: a video analysis of 464 head injury events to inform proposed injury prevention strategies. Br J Sports Med..

[2] Manley G, Gardner AJ, Schneider KJ, Guskiewicz KM, Bailes J, Cantu RC (2017). A systematic review of potential long-term effects of sport-related concussion. Br J Sports Med..

[3] Tucker R, Raftery M, Verhagen E (2016). Injury risk and a tackle ban in youth Rugby Union: reviewing the evidence and searching for targeted, effective interventions. A critical review. Br J Sports Med..

[4] Tucker R, Raftery M, Fuller GW, Hester B, Kemp S, Cross MJ (2017). A video analysis of head injuries satisfying the criteria for a head injury assessment in professional Rugby Union: a prospective cohort study. Br J Sports Med..

[5] Cross MJ, Tucker R, Raftery M, Hester B, Williams S, Stokes KA (2019). Tackling concussion in professional rugby union: a case-control study of tackle-based risk factors and recommendations for primary prevention. Br J Sports Med..

[6] Hendricks S, O'Connor S, Lambert M, Brown J, Burger N, Mc Fie S (2015). Contact technique and concussions in the South African under-18 Coca-Cola Craven Week Rugby tournament. Eur J Sport Sci..

[7] McIntosh AS, McCrory P, Finch CF, Wolfe R (2010). Head, face and neck injury in youth rugby: incidence and risk factors. Br J Sports Med..

[8] Palmer-Green DS, Stokes KA, Fuller CW, England M, Kemp SPT, Trewartha G (2013). Match injuries in English youth academy and schools rugby union: an epidemiological study. Am J Sports Med..

[9] Chéradame J, Piscione J, Carling C, Guinoiseau JP, Dufour B, Jacqmin-Gadda H (2021). Incidence and risk factors in concussion events: a 5-season study in the French top 14 rugby union championship. Am J Sports Med..

[10] Quarrie KL, Hopkins WG (2008). Tackle injuries in professional Rugby Union. Am J Sports Med..

[11] Burger N, Lambert MI, Viljoen W, Brown JC, Readhead C, Hendricks S (2016). Tackle technique and tackle-related injuries in high-level South African Rugby Union under-18 players: real-match video analysis. Br J Sports Med..

[12] Tierney GJ, Simms CK (2017). The effects of tackle height on inertial loading of the head and neck in Rugby Union: a multibody model analysis. Brain Inj..

[13] Sobue S, Kawasaki T, Hasegawa Y, Shiota Y, Ota C, Yoneda Y (2018). Tackler's head position relative to the ball carrier is highly correlated with head and neck injuries in rugby. Br J Sports Med..

[14] Tierney GJ, Denvir K, Farrell G, Simm CK (2018). Does player time-in-game affect tackle technique in elite level rugby union?. J Sci Med Sport..

[15] Tierney GJ, Richter C, Denvir K, Simms CK (2018). Could lowering the tackle height in rugby union reduce ball carrier inertial head kinematics?. J Biomech..

[16] Tierney GJ, Simms CK (2018). Can tackle height influence head injury assessment risk in elite rugby union?. J Sci Med Sport..

[17] Stokes KA, Locke D, Roberts S, Henderson L, Tucker R, Ryan D (2021). Does reducing the height of the tackle through law change in elite men's rugby union (The Championship, England) reduce the incidence of concussion? A controlled study in 126 games. Br J Sports Med..

[18] Fuller CW, Ashton T, Brooks JHM, Cancea RJ, Hall J, Kemp SPT (2010). Injury risks associated with tackling in rugby union. Br J Sports Med..

[19] Davidow D, Quarrie K, Viljoen W, Burger N, Readhead C, Lambert M (2018). Tackle technique of rugby union players during head impact tackles compared to injury free tackles. J Sci Med Sport..

[20] Hendricks S, Till K, den Hollander S, Savage TN, Roberts SP, Tierney G (2020). Consensus on a video analysis framework of descriptors and definitions by the Rugby Union video analysis consensus group. Br J Sports Med..

[21] Williams S, Trewartha G, Kemp S, Stokes K (2013). A meta-analysis of injuries in senior men's professional Rugby Union. Sports Med..

[22] Tierney GJ, Lawler J, Denvir K, McQuilkin K, Simms CK (2016). Risks associated with significant head impact events in elite rugby union. Brain Inj..

