# Supplementary material for: Risk Factors for Concussion in Under 18, Under 22 and Professional Men's Rugby Union: A Video Analysis of 14,809 Tackles
Source: Sports Med Open. 2023 Oct 14;9:95. doi: 10.1186/s40798-023-00642-z (PMC10576729; doi:10.1186/s40798-023-00642-z)
Supplement: Supplementary file 1 — Additional file 1: Table S1. Description of tackle characteristic and factors analysed in this study. Table S2. Participant demographics. [file 40798_2023_642_MOESM1_ESM.docx]

**SUPPLEMENTARY MATERIALS**

**Title: Risk factors for concussion in Under 18, Under 22, and professional men's rugby union: A video analysis of 14809 tackles**

**journal name: Sports Medicine - Open**

**Authors: Takayuki Kawasaki^1^, Yuta Kawakami^2^, Shuko Nojiri^3^, Yoshinori Hasegawa^1^, Manabu Kuroki^4^, Shogo Sobue^1^, Kenta Shibuya^1^, Yuji Takazawa^1,5^, Muneaki Ishijima^1^**

**1 Department of Orthopaedics Surgery, Faculty of Medicine, Juntendo University, Tokyo, Japan.**

**2 Department of Mathematics, Physics, Electrical Engineering and Computer Science, Graduate School of Engineering Science, Yokohama National University, Kanagawa, Japan.**

**3 Department of Medical Technology Innovation Center, Juntendo University, Tokyo, Japan.**

**4 Division of Intelligent Systems Engineering, Faculty of Engineering, Yokohama National University, Kanagawa, Japan.**

**5 Faculty of Health and Sports Science, Juntendo University, Chiba, Japan.**

**Correspondence:**

**A/Prof. Takayuki Kawasaki**

**Department of Orthopaedic Surgery**

**Juntendo University Faculty of Medicine**

**2-1-1, Bunkyo, Tokyo, 113-8421, Japan**

**TEL: +81-3-3813-3111**

**FAX: +81-3-5802-3428**

**Email: k-saki@luck.ocn.ne.jp**

[**https://orcid.org/0000-0003-3306-8506**](https://orcid.org/0000-0003-3306-8506)

**Supplementary Table**

**Table S1. Description of tackle characteristic and factors analysed in this study**

| **Term** | **Description** |
| --- | --- |
| **Tackle Characteristics** | |
| ***Head placement on the side of the ball-carrier*** | |
| Head-in-front tackle | Tackler's head is placed forward and impedes the ball carrier's movement. |
| Correct head tackle | Tackler's head is not placed forward, (e.g. to the side and not impedes the ball carrier's movement). |
| ***Direction*** | |
| Front-on | Tackler makes contact on the front of the ball carrier |
| Side-on | Tackler makes contact on the side of the ball carrier |
| Behind | Tackler makes contact on the ball carrier from behind |
| ***Tackle Height (Tackler’s first point of contact on the ball-carrier)*** | |
| Chest | Tackler makes contact on the chest (arm pit to shoulder) or above the chest (e.g. smother) of the ball carrier |
| Waist (Torso) | Tackler makes contact on the waist of the ball carrier |
| Below the hip | Tackler makes contact below the hip (shorts line) of the ball carrier |
| ***Tackle type*** | |
| Shoulder | First contact is made by the tackler's shoulder on the ball carrier. |
| Arm | First contact is made by the tackler's arm on the ball carrier. |
| Smother | Tackler wraps both arms around the ball carrier to trap the ball. |
| **Other Factors** | |
| ***Player level*** | |
| U18 | Participants who are high-school students and under 18 years old |
| U22 | Participants who are university/college students and under 22 years old |
| Elite | Participants who are professional Rugby Union players |
| ***Positional mismatch*** | Tackle is made by players from different positions (i.e. forwards vs backs) |
| ***Time of the match*** | |
| 1st quarter | The first 20 min of game time in the 40 min of the 1st half |
| 2nd quarter | The second 20 min of game time in the 40 min of the 1st half |
| 3rd quarter | The first 20 min of game time in the 40 min of the 2nd half |
| 4th quarter | The second 20 min of game time in the 40 min of the 2nd half |
| ***Player position*** | |
| Forwards | A player who is allocated to one of the eight forward positions (numbered 1-8) |
| Backs | A player who is allocated to one of the seven back positions (9-15) |
| ***Player area*** | |
| Area 1 | The defending area between the try-line and the 22m line |
| Area 2 | The defending area between the 22m line and the 10m line |
| Area 3 | The area between defending the 10m line and offending the 10m line |
| Area 4 | The offending area between the 10m line and the 22m line |
| Area 5 | The offending area between the 22m line and the try-line |
| ***Score difference*** | The score difference at the point of a tackle: the score of the tackler's team - the score of the ball carrier's team; a positive score indicates the tackler on the winning side. |
| ***Final score difference*** | The final score difference at the point of a tackle |
| ***Duration 1*** | The duration from a starting phase (scrum, lineout, maul, ruck etc.) until the next tackle |
| ***Duration 2*** | The duration between the moment the player last obtained the ball and the time of being tackled. |
| ***Number of phases*** | Number of phases (a series of passes and plays) from a set-piece until the tackle |

**Table S2. Participant demographics**

|  | **U18** | | | **U22** | | | **Elite** | | |
| --- | --- | --- | --- | --- | --- | --- | --- | --- | --- |
| Player position | Forwards | backs | Total | Forwards | Backs | Total | Forwards | Backs | Total |
| **Number of players** | 320 | 280 | 600 | 320 | 280 | 600 | 320 | 280 | 600 |
| **Age (years)** | 17.4 (0.7) | 17.3 (0.7) | 17.4 (0.7) | 21.0 (1.2) | 20.7 (1.1) | 20.8 (1.1) | 28.1 (3.6) | 27.3 (3.3) | 27.8 (3.5) |
| **Height (cm)** | 175.2 (6.3) | 172.6 (7.3) | 174.0 (6.9) | 180.1 (6.3) | 175.2 (5.6) | 177.8 (6.5) | 185.1 (10.4) | 178.6 (6.1) | 182.0 (9.3) |
| **Weight (kg)** | 88.5 (11.5) | 74.1 (7.5) | 81.8 (12.2) | 101.4 (9.3) | 83.1 (8.1) | 92.9 (12.6) | 108.2 (9.5) | 88.7 (8.0) | 99.1 (13.1) |
| **BMI (kg/m^2^)** | 28.9 (3.8) | 25.1 (5.3) | 27.1 (5.0) | 31.3 (2.7) | 27.0 (1.7) | 29.3 (3.1) | 31.9 (5.6) | 27.8 (1.4) | 30.0 (4.7) |
| **Race (%)** |  |  |  |  |  |  |  |  |  |
| **Asian** | 319 (99.7%) | 279 (99.6%) | 598 (99.7%) | 291 (90.9%) | 265 (94.6%) | 556 (92.7%) | 190 (59.4%) | 170 (60.7%) | 360 (60.0%) |
| **Islander** | 1 (0.3%) | 1 (0.4%) | 2 (0.3%) | 19 (5.9%) | 9 (3.2%) | 28 (4.7%) | 48 (15.0%) | 49 (17.5%) | 97 (16.2%) |
| **White** | 0 (0.0%) | 0 (0.0%) | 0 (0.0%) | 10 (3.1%) | 6 (2.1%) | 16 (2.7%) | 82 (25.6%) | 61 (21.8%) | 143 (23.8%) |

Mean (standard deviation, SD) are shown unless stated otherwise.

The replacement players who were “on the bench” are not included in the number of players indicated.
